# The Subscapularis Muscle: A Proposed Classification System

**DOI:** 10.1155/2021/7450000

**Published:** 2021-12-11

**Authors:** Nicol Zielinska, R. Shane Tubbs, Andrzej Borowski, Michał Podgórski, Łukasz Olewnik

**Affiliations:** ^1^Department of Anatomical Dissection and Donation, Medical University of Lodz, Poland; ^2^Department of Neurosurgery, Tulane University School of Medicine, New Orleans, LA, USA; ^3^Department of Neurosurgery and Ochsner Neuroscience Institute, Ochsner Health System, New Orleans, LA, USA; ^4^Department of Anatomical Sciences, St. George's University, Grenada; ^5^Department of Neurology, Tulane University School of Medicine, New Orleans, LA, USA; ^6^Department of Structural and Cellular Biology, Tulane University School of Medicine, New Orleans, LA, USA; ^7^Orthopaedics and Pediatric Orthopaedics Department, Medical University of Lodz, Poland; ^8^Polish Mothers' Memorial Hospital Research Institute, Lodz, Poland

## Abstract

**Background:**

On the basis of the available literature, we proposed the hypothesis that the number of muscle bellies is morphologically constant. The main purpose of this study was to examine the morphological variability of the SM and to create a new classification of it based on number of muscle bellies.

**Methods:**

Sixty-six adult cadavers of Central European population (45 females, 21 males) were obtained and fixed in 10% formalin before examination.

**Results:**

The SM was found in all 66 specimens (45 females, 21 males, 31 left and 35 right sides). After meticulous dissection, we distinguished nine types on the basis of number of bellies. Type I was characterized by single belly and occurred in 1.5%. Type II had a double belly and was present in 3%. Type III, the most common type, occurring in almost 32% of the studied population, had three bellies. The frequency of type IV, characterized by four bellies, was also high, just over 30%. The following types were less frequent: type V with five bellies (18.2%), type VI with six bellies (7.6%), type VII with seven bellies (3%), type VIII with eight bellies (1.5%), and type IX with nine bellies (3%). All of the types had origin on the anterior surface of the scapula.

**Conclusions:**

The SM is morphologically variable in the number of its bellies. Evolutionary changes are probably the reason. The most common type was the SM with three bellies, in line with Larson's model of the division of the SM into three parts. Subsequent studies should be carried out based on MRI or ultrasonography examination to confirm if it is possible to show all types (presented in this study) among group of patients during MRI.

## 1. Introduction

The subscapularis muscle (SM) is the most powerful and largest muscle in the rotator cuff. Its characteristic feature is that its function is internal rotation; the other muscles that make up the rotator cuff (teres minor, supraspinatus, and infraspinatus) are responsible for external rotation [1, 2]. The SM comprises muscular and tendinous parts. The muscular part has origin on the anterior part of the scapula, called the subscapular fossa [2]. Importantly, this muscle occupies the entire fossa, so it is recognized as the largest muscle attached to the scapula [3]. The distal attachment, which is a terminal fragment of the tendinous part, is situated on the superior part of the humerus (in most cases, the lesser tuberosity). The upper subscapularis nerve (USN) and the lower subscapularis nerve (LSN) are responsible for innervating the SM. They both arise from the posterior cord of the brachial plexus and receive contributions from C5 and C6. The LSN is more morphologically variable than the USN [3]. The subscapular artery (arising as the first branch from the third part of the axillary artery) is commonly recognized as the main supplier of blood to the SM [4], though some research indicates that the anterior humeral circumflex artery is mainly responsible for this [3, 5].

As mentioned above, the main function of the SM is internal rotation, but in certain positions, it can work as adductor and extensor [6]. It is also responsible for limiting the superior migration of the humeral head and maintaining its anteroposterior position, making it particularly important in providing both dynamically and passively for glenohumeral joint stability [3].

The SM is morphologically highly variable in both origin and insertion [1, 6–8]. The proximal attachment is located in the subscapular fossa, but in some cases, the origin is fused with another muscle such as the latissimus dorsi or teres major [3]. In turn, the distal attachment can vary in number of tendons and placement of insertion [1]. Zielinska et al. [6] created a new classification system of the subscapularis tendon, distinguishing four types on the basis of number of tendons. In most cases, the distal attachment is on the lesser tuberosity, but the greater tubercle, bicipital groove, or another structure on the superior part of the humerus can serve instead [6]. An accessory subscapularis muscle is very rare; it was described for example by Zielinska et al. [1], Yoshinaga et al. [7], and Krause and Youdas [8].

The SM is clinically important [1, 3, 6]. For example, it must usually be separated in open shoulder surgery, or the tendinous part will be destroyed [6]. Accurate muscle classification based on Zielinska et al. [6] facilitates this operation. Open shoulder surgery can entail complications such as nerve or artery injury, potentially causing weakening or loss function of the SM [3]. The subscapularis nerve or artery can also be compressed by an additional structure such as an accessory subscapularis muscle. In this case there is a risk of quadrilateral space syndrome, muscular weakness and atrophy, or wrist drop [1].

The morphological variations and additional structures of the SM have been described in a few articles, but there has been no work on evaluating the muscle bellies. The main aim of our present work is to classify SM types according to the number of bellies. Another goal is to make morphometric measurements of the SM and present its statistically significant features. The SM is commonly regarded as a single whole muscle, not divided into individual bellies, or divided only into three parts (superior, middle, and inferior) without reference to individual bellies. We initially hypothesize that the number of bellies is morphologically constant.

## 2. Materials and Methods

Sixty-six upper limbs (45 females, 21 males, 31 left and 35 right sides) were obtained from adult cadavers of Central European population and fixed in 10% formalin before examination. The mean age “at death” of the cadavers was 73.7 years (48-96). The cadavers were the property of the Department of Anatomical Dissection and Donation, Medical University of Lodz, Poland, following donation to the university anatomy program. We confirm that all methods were performed in accordance with the relevant guidelines and regulations.

All dissections of the shoulder and arm areas followed a preestablished protocol [1, 6, 9–12]. Upper limbs with visible signs of possible surgical intervention were not included in the group of limbs examined.

The first stage of dissection was to remove the skin and superficial fascia of the medial side of the arm and shoulder region. The lateral, medial, and posterior cords of the brachial plexus were then visualized. The next step included visualization of the biceps brachii and coracobrachialis muscles; structures anterior to the SM such as nerves, arteries, and veins were then gently cleansed. The next step depended on removal of the fascia covering the SM. The tendinous and muscular parts of SM were then cleaned and checked in the medial direction. The number of bellies and their possible bifurcation were noted. Following this, all structures were thoroughly cleaned.

**Figure 1:**
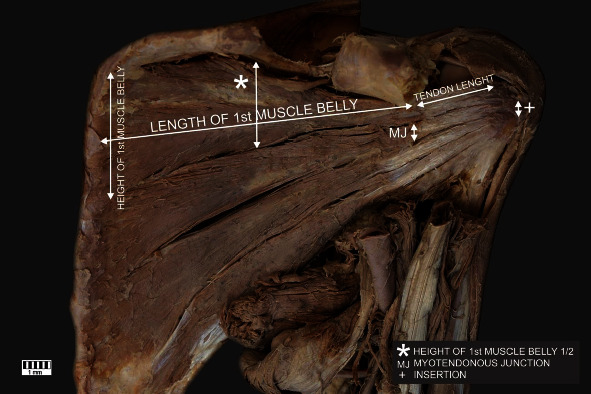
Morphological measurements of the subscapularis muscle.

Upon dissection, the following were assessed:

- The number of bellies of the SM
- Morphometric measurements of the SM
- Morphometric measurements of the skeletal system associated with shoulder girdle (Figure 1)

When dissecting the SM:

- Special attention should be paid during removal of the fascia because there is sometimes an ASM [1]
- Particular attention should be paid to cleansing the head of the long biceps brachii because there is a risk that the SM tendons will be cut

### 2.1. Creating a Classification

The number of bellies was counted on the basis of the number of tendon cores between the bellies and the fascia. Importantly, the muscle bellies were separated from each other by a fascia, which penetrated the scapula.

An electronic digital caliper (Mitutoyo Corporation, Kawasaki-shi, Kanagawa, Japan) was used for all measurements. Each measurement was made twice, with an accuracy of 0.1 mm. The study protocol was approved by the Bioethics Committee of the Medical University of Lodz (resolution RNN/1337/20/KE). The cadavers belonged to the Department of Anatomical Dissection and Donation of the Medical University of Lodz, Poland.

### 2.2. Statistical Analysis

Statistica 13.1 software (StatSoft Polska, Cracow, Poland) was used for statistical analyses. The chi [2] test was used to assess the association between types of muscle belly and sex/body sides. The Shapiro-Wilk test was used to check the normality of distribution. As the data were not normally distributed, nonparametric tests, the Mann-Whitney test, and Kruskal-Wallis test by ranks with dedicated post hoc test were used to compare measurements between SM belly types.

A *p* value lower than 0.05 was considered significant, with Bonferroni correction for multiple testing. The results are presented as mean and standard deviation unless otherwise stated.

## 3. Results

**Figure 2:**
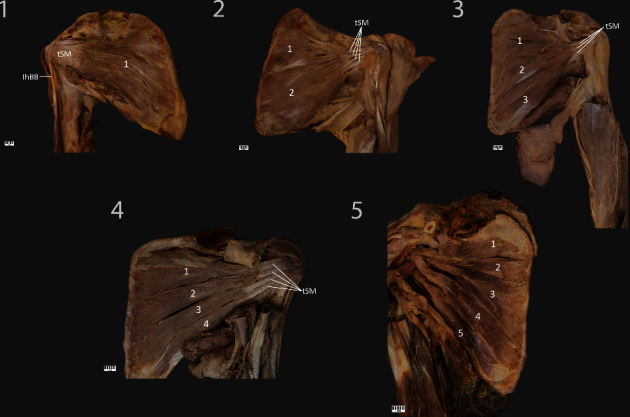


The SM was present in all 66 specimens. There were many muscle belly variants, and the following types were differentiated on the basis of meticulous dissection:

Type I: this type is characterized by a single belly; its proximal attachment is located on the anterior surface of the scapula (subscapular fossa). The muscular part is continued as a tendinous part. In our research, the case representing this type ended in a single tendon. This type was found in one upper limb (1.5%) (Figure 2(1)).

Type II: this type is characterized by a double belly; the proximal attachment is located on the anterior surface of the scapula (subscapular fossa). The muscular part is continued as a tendinous part; the site of insertion and number of tendons can vary. In our research, the cases representing this type ended in three or four tendons. This type was found in two upper limbs (3%) (Figure 2(2)).

Type III: this type is characterized by three bellies; the proximal attachment is located on the anterior surface of the scapula (subscapular fossa). The muscular part is continued as a tendinous part; the site of insertion and number of tendons can vary. In our research, all cases representing this type ended in one, two, or three tendons. It was found in twenty-one upper limbs (31.9%) (Figure 2(3)).

Type IV: this type is characterized by four bellies; the proximal attachment is located on the anterior surface of the scapula (subscapular fossa). The muscular part is continued as a tendinous part; the site of insertion and number of tendons can vary. In our research, all cases representing this type ended in one, two, three, four, or five tendons. This type was found in twenty upper limbs (30.3%) (Figure 2(4)).

Type V: this type is characterized by five bellies; the proximal attachment is located on the anterior surface of the scapula (subscapular fossa). The muscular part is continued as a tendinous part; the site of insertion and number of tendons can vary. In our research, all cases representing this type ended in one, two, five, or six tendons. This type was found in twelve upper limbs (18.2%) (Figure 2(5)).

**Figure 3:**
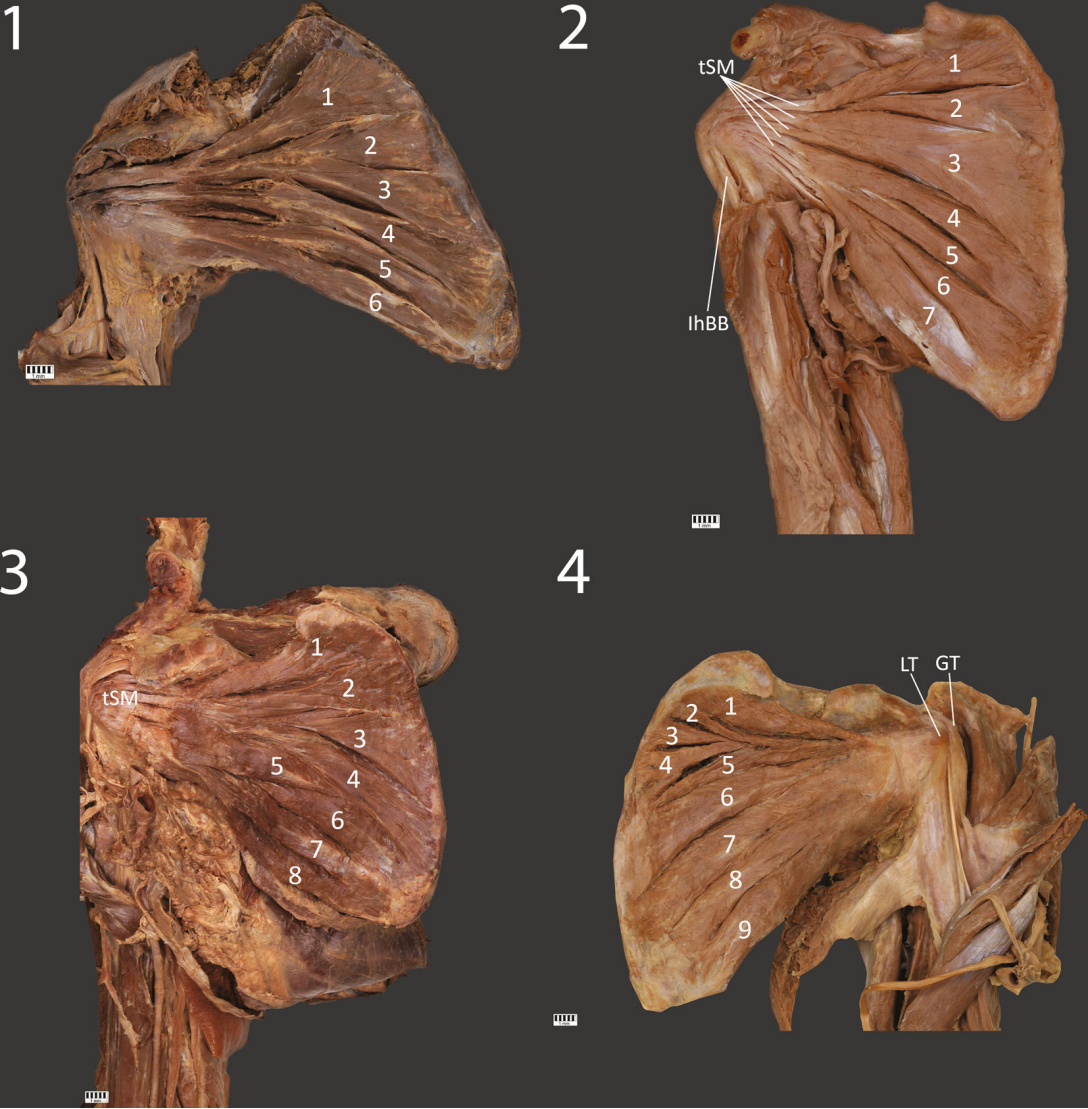


Type VI: this type is characterized by six bellies; the proximal attachment is located on the anterior surface of the scapula (subscapular fossa). The muscular part is continued as a tendinous part; the site of insertion and number of tendons can vary. In our research, all cases representing this type ended in five or six tendons. This type was found in five upper limbs (7.6%) (Figure 3(1)).

Type VII: this type is characterized by seven bellies; the proximal attachment is located on the anterior surface of the scapula (subscapular fossa). The muscular part is continued as a tendinous part; the site of insertion and number of tendons can vary. In our research, both cases representing this type ended in seven tendons. This type was found in two upper limbs (3%) (Figure 3(2)).

Type VIII: this type is characterized by eight bellies; the proximal attachment is located on the anterior surface of the scapula (subscapular fossa). The muscular part is continued as a tendinous part; the site of insertion and number of tendons can vary. In our research, the case representing this type ended in four tendons. This type was found in one upper limb (1.5%) (Figure 3(3)).

Type IX: this type is characterized by nine bellies; the proximal attachment is located on the anterior surface of the scapula (subscapular fossa). The muscular part is continued as a tendinous part; the site of insertion and number of tendons can vary. In our research, both cases representing this type ended in eight tendons. This type was found in two upper limbs (3%) (Figure 3(4)).

The distribution of muscle bellies according to sex and body side is presented in Table 1. There were no significant sex (*p* = 0.0712) or body side (*p* = 0.8834) differences.

Morphometric parameters are compared among SM types in Table 2. Using Bonferroni correction and post hoc analysis, only the height of the first muscle belly length in type I was greater than in all other types, and the height half-way along the third muscle belly length was greater in types III and IV than that in types VII, VIII, and IX.

## 4. Discussion

The SM is commonly regarded as a single muscle without division into individual bellies, or with division into three parts (superior, middle, and inferior) without distinction among individual bellies. In our research, we found cases with 1-9 muscle bellies. This allowed us to create a new classification, distinguishing nine types of SM based on the number of bellies. Moreover, these results refuted our preliminary hypothesis that the number of bellies is morphologically constant. The differentiation could have an evolutionary basis.

The SM has shrunk between phylogenetically plesiomorphic hominoids and modern humans [13]. Larson [14] noticed that among both chimpanzees and hominoids, the SM comprised three parts: superior, middle, and inferior. Depending on the position of the upper limb, each of these parts acts individually, helping to control the rotator state of the humerus [15]. The inferior and the middle parts are active during arm-swinging during the first half of the medial rotation. During climbing, the superior part is active; the other parts are also active but to a lesser extent [14, 15].

In African apes, medial rotation of the humerus is particularly significant during knuckle-walking because it compensates for the force acting on the glenohumeral joint and stabilizes it [15–19]. The SM does not stabilize the glenohumeral joint during arm-swinging by chimpanzees. It is active among hylobatids during the support phase of arm-swinging but fulfills a rotatory rather than a stabilizing function. We can conclude that it participates more in free arm movements in hylobatids than in chimpanzees [17, 20]. However, the SM has the dominant role during the first stage of vertical climbing [20, 21]. There is an expectation that the SM should be larger in climbing species than among species without climbing skills [21]. In fact, both great apes and hylobatids have wide lateral enlargement of the anterior surface of the scapula, but the SM is comparatively wider among hylobatids. Among hominids, this is an adaptive change during brachiation, allowing for proper positioning of the upper limb (which might seem difficult because of low degree of humeral head torsion, putting the elbow joint in lateral position) [19, 21]. Modern humans have a smaller lateral expansion of the SM relative to body size, presumably because of the reduced need for climbing [21].

Combining the above facts, we noticed some dependences. We assume that the number of bands increased throughout evolution: at the beginning, the SM was not divided into distinct bands, resulting in high stabilization in a four-legged gait (the work of the SM entails only arm-swinging during first half of the medial rotation). Nonhuman hominoids started climbing, so it became necessary to divide the SM into more parts. Maybe division into more than three bands allows the SM to be engaged in a wider range of movements. In our study, the number of bands ranged from one to nine, in line with the foregoing assumptions.

In type I, one belly is responsible for performing every function of the SM, so we can only suppose that the movements are not very precise, though the stabilization is really high. Type II, in which the SM has two bellies, presents a similar situation. The subsequent types have increasing numbers of bellies what probably was connected with increasing precision of movements and decreasing stabilization. Moreover, the division into distinct smaller bellies could weaken the SM overall.

The frequency distribution of the types distinguished in our research is interesting. On this basis, we proposed another hypothesis: not only has there been evolutionary progression (increasing the number of bellies) but also there has been evolution regression (to optimize the number of bellies).

The most common type was type III, with three bellies, in line with Larson's model of the division of the SM into three parts. Muscles with four (type IV) or five (type V) bellies were also frequent. So, to clarify our additional assumptions, we included these types in one group of muscles with an optimal number of bellies. Continuing, we created another two potential groups: with fewer bellies and with more bellies. The group of muscles with fewer bellies includes type I, which was found in only one cadaver, and type II, which occurred twice among the population researched. The group with most bellies includes type VI, which was found in five cases, type VII in two, type VIII in one, and type IX in two.

Summarizing our assumptions above (identifying three groups to elucidate our way of thinking) and the results of our research, the overall prevalence of the group of muscles with an optimal number of bellies was 80.4%; that for the group of muscles with fewer bellies was 4.5%; that for the group of muscles with more bellies was 15.1%.

As mentioned above, fewer bellies potentially are associated with high stabilization and great SM strength. In contrast, more bellies probably are associated with high precision of movements. So the optimal number of bellies seems to be a balance among good stabilization, optimal precision, and preservation of strength.

However, this does not necessarily preclude our first hypothesis that the number of bands increased throughout evolution. It is highly probable that evolution was driven by the need for the greatest possible precision of the SM. When there was a tendency to develop more bellies (as in types VI-IX), this evolutionary tendency became disadvantageous and entailed decreased stabilization of the shoulder girdle and decreased strength. We suppose that evolutionary regression began at this point, optimizing the number of bellies (types III-V) to provide good stabilization, satisfactory precision of movements, and sufficient muscle strength. This seems to corroborate our second hypothesis, that not only evolutionary progress (increasing the number of bellies) but also evolutionary regression (optimizing the number of bellies) occurred.

Studies of ontogenesis suggest that the SM is formed from three primitive muscle masses innervated by different nerves during the early stage of embryonic development [1, 3]. However, it is interesting to ask which ontogenetic stages are changed so that different numbers of muscle bellies are formed. This has not been studied.

After confirming the significance of different number of bellies in the evolution of the SM, we wondered whether this is clinically significant, as in the different numbers of subscapularis tendons [6]. The division of the tendinous part of the SM into distinct tendons helps to prevent tearing of the structure and loss of function, allowing nonoperative treatment alone to suffice. A wide, nondivided tendon is more predisposed to tear completely, usually requiring more invasive treatment [22–24]. However, we observed no similar relationship for the different numbers of bellies, so we continued to search for its clinical significance.

Another example to serve as a reference is the occurrence of an additional belly as exemplified by the coracobrachialis muscle (CBL) [25, 26]. The additional head of the CBL can compress the median and musculocutaneous nerves, which is associated with weakness or loss of function of the muscle in the anterior compartment of the arm and forearm [27]. However, the location of the SM does not predispose it to compress any structure. The muscular part (which consists of bellies) fills the entire subscapular fossa [28], and this muscle is the deepest structure on the anterior surface of the scapula (beyond it, the tendinous part begins). That is why the difference in numbers of bellies does not entail an increased risk of pressure on the nerves, arteries, or other muscles.

However, in continuing our search for the clinical significance of the different numbers of bellies, we refer to the function of the SM. As mentioned above, the SM is responsible for providing anterior glenohumeral joint stability [3]. It is involved in glenohumeral placing, particularly in avoiding the superior shearing force of the deltoid muscle during abduction. Thanks to this, the deltoid muscle retains its mechanical predominance, rather than merely driving the humeral head into the acromion for the duration of vigorous abduction [13, 29, 30]. One study showed that the activity of the SM is maximized in abduction around 60–80 degrees because it works as a structure resisting humeral migration [13]. The SM is also involved in limiting the anteroposterior translation of the humeral head, so it not only limits superior migration of the humeral head but also helps it to maintain its anteroposterior position [29, 31, 32].

In the first part of this discussion, we assumed that the increasing number of bellies is probably associated with decreasing stabilization, so it could be clinically significant. There is a relationship showing that an SM comprising one wide belly (type I in our research) provides much more stabilization of the glenohumeral joint than one comprising many bellies (for example, type VIII or type IX in our research). We can distinguish three types of glenohumeral dislocation: anterior, posterior, and inferior [22].

Anterior glenohumeral dislocation is usually caused by a blow to an abducted, externally rotated, and extended extremity. Posterior dislocation follows a strong impact to the anterior shoulder and axial loading of the adducted and internally rotated upper limb, or it can result from an electric shock or convulsions causing SM contraction [33]. Owing to its function (maintaining the anteroposterior position of the humeral head), the SM prevents glenohumeral dislocation. When the SM comprises few bellies, this prevention is enhanced.

Another pathology connected with the humeral head is a superior subluxation, which entails superior migration of the humeral head [34]. As mentioned above, the SM is also responsible for limiting such migration. The situation is analogous to that described in the previous paragraph. A strong, single, wide belly is potentially better able to prevent superior humeral head migration. An SM with a multiband origin (e.g., type VIII or IX in our research) also may fulfill this function, but less efficiently.

Summing up, the previous hypothesis that number of bellies of the SM is morphologically constant was refuted. Our results showed that we can divide this muscle into nine types according to the number of bellies. However, if future research finds muscles with more bellies, this classification should be amended. We suggest that the basis for such differentiation is evolutionary. It is worth mentioning that not only evolutionary progression but also evolutionary regression could have taken place in response to the conflicting needs for optimal stabilization, satisfactory precision of movements, and sufficient SM strength. Different numbers of bellies could be clinically significant in humeral head dislocation cases.

This study has some limitations. A larger sample size would have been desirable; the small size (*n* = 66) was sufficient to demonstrate the morphological variability of bellies of the SM for the first time. In addition, the study population was recruited from a specific population of people who had lived most of their lives in the region around Lodz, Poland. Therefore, more extensive studies are needed to determine whether the observed division is matched in larger populations. In addition, the sample size was not calculated; last but not least limitation is that the ultrasonography and MRI examination have not been carried out; however, this study is one of the largest to date on morphological variations of the proximal attachments of the SM. It is the first study of this magnitude to lead to a completely new classification of SM muscle bellies. Such a systematic classification will be valuable for improving the results of future interventions in the arm and shoulder area.

## 5. Conclusions

The SM is morphologically variable in the number of its bellies. Evolutionary changes are probably the reason. The most common type was the SM with three bellies, in line with Larson's model of the division of the SM into three parts. Subsequent studies should be carried out based on MRI or ultrasonography examination to confirm if it is possible to show all types (presented in this study) among group of patients during MRI.

## Figures and Tables

**Table 1:** Distribution of muscle bellies according to sex and body side.

Muscle type	Sex [*n* (%)].		Body side [*n* (%)].	
	Female	Male	Right	Left
I	1 (2.2)	0 (0.0)	0 (0.0)	1 (3.2)
II	2 (4.4)	0 (0.0)	1 (2.9)	1 (3.2)
III	17 (37.8)	4 (19.1)	12 (34.3)	9 (23.0)
IV	15 (33.3)	5 (23.8)	11 (31.4)	9 (23.0)
V	6 (13.3)	6 (28.6)	5 (14.3)	7 (22.6)
VI	2 (4.4)	3 (14.3)	3 (8.6)	2 (6.5)
VII	0 (0.0)	2 (9.5)	1 (2.9)	1 (3.2)
VIII	0 (0.0)	1 (4.8)	1 (2.9)	0 (0.0)
IX	2 (4.4)	0 (0.0)	1 (2.9)	1 (3.2)

**Table 2:** Comparison of morphometric parameters according to subscapularis muscle type.

Parameter	Muscle belly type									*p* value
	I	II	III	IV	V	VI	VII	VIII	IX	
Humerus length	251.21 (-)	255.90 (0.01)	250.46 (16.11)	254.23 (30.03)	259.02 (30.33)	271.55 (25.68)	275.89 (0.46)	265.32 (-)	271.16 (0.07)	0.4412
Distance between superior and inferior angle	162.35 (-)	150.85 (0.18)	143.00 (12.63)	148.48 (22.49)	161.13 (20.07)	157.73 (9.71)	164.88 (0.33)	164.23 (-)	154.25 (0.35)	0.0853
Distance between inferior angle and lesser tubercle	195.04 (-)	154.17 (0.22)	161.76 (13.55)	158.86 (12.68)	179.47 (21.32)	166.43 (18.84)	194.90 (0.41)	195.78 (-)	172.25 (0.10)	0.0181
Distance between inferior angle and greater tubercle	199.98 (-)	176.71 (0.71)	176.07 (13.22)	176.08 (14.89)	179.89 (34.50)	190.89 (11.86)	204.33 (0.33)	201.21 (-)	186.66 (0.50)	0.0957
Distance between superior angle and lesser tubercle	121.34 (-)	99.71 (0.71)	112.74 (12.90)	119.05 (11.50)	116.93 (16.63)	108.27 (10.06)	124.76 (0.17)	123.32 (-)	119.10 (0.27)	0.2606
Distance between superior angle and greater tubercle	127.65 (-)	120.55 (0.64)	121.88 (27.73)	132.97 (12.16)	126.60 (17.21)	122.56 (13.01)	140.72 (0.25)	128.32 (-)	127.68 (0.66)	0.3850
Length of the 1^st^ muscle belly	116.81 (-)	85.58 (0.04)	87.19 (12.64)	84.04 (14.13)	88.77 (11.32)	91.18 (10.98)	80.87 (0.01)	76.21 (-)	78.14 (0.04)	0.4141
Height of the 1^st^ muscle belly	121.69 (-)	11.29 (0.02)	37.07 (7.35)	28.99 (9.59)	38.22 (14.21)	16.47 (6.71)	17.94 (0.10)	16.77 (-)	17.69 (0.04)	0.0000
Height of the 1^st^ muscle belly at 1/2 length	71.03 (-)	15.97 (0.06)	24.65 (6.59)	19.49 (7.29)	20.00 (4.49)	13.53 (8.56)	14.97 (0.07)	14.33 (-)	100.66 (0.01)	0.0064
Length of the 2^nd^ muscle belly		82.37 (0.06)	98.51 (24.34)	96.81 (16.07)	106.66 (16.11)	77.44 (44.46)	98.89 (0.02)	95.21 (-)	12.46 (0.12)	0.1333
Height of the 2^nd^ muscle belly		28.55 (0.01)	38.12 (10.40)	29.75 (9.98)	26.35 (7.18)	22.37 (14.56)	20.71 (0.07)	19.21 (-)	22.19 (0.18)	0.0049
Height of the 2^nd^ muscle belly at 1/2 length		26.84 (0.52)	22.45 (6.02)	19.60 (6.90)	17.81 (8.32)	15.45 (9.77)	14.32 (0.01)	14.12 (-)	9.92 (0.25)	0.0526
Length of the 3^rd^ muscle belly		104.96 (0.08)	122.15 (16.06)	110.26 (18.00)	121.64 (17.95)	113.48 (17.84)	103.50 (0.09)	100.21 (-)	116.78 (0.03)	0.1561
Height of the 3^rd^ muscle belly		49.88 (0.47)	35.55 (7.72)	29.11 (8.67)	37.22 (24.99)	21.80 (16.15)	19.13 (0.11)	18.21 (-)	23.19 (0.34)	0.0403
Height of the 3^rd^ muscle belly at 1/2 length		23.46 (0.35)	24.96 (5.02)	17.73 (5.62)	15.45 (4.88)	13.78 (7.83)	12.94 (0.09)	12.11 (-)	8.20 (0.01)	0.0001
Length of the 4^th^ muscle belly		109.18 (0.06)		120.92 (16.06)	126.04 (15.18)	117.63 (8.76)	113.34 (0.13)	112.32 (-)	115.73 (0.84)	0.5402
Height of the 4^th^ muscle belly		26.79 (0.03)		30.85 (4.84)	25.42 (7.86)	17.89 (11.79)	15.27 (0.08)	14.56 (-)	15.74 (0.43)	0.0108
Height of the 4^th^ muscle belly in 1/2 length		21.84 (0.08)		22.35 (6.00)	19.36 (4.83)	11.24 (6.23)	11.89 (0.18)	11.21 (-)	8.58 (0.05)	0.0052
Length of the 5^th^ muscle belly					123.96 (8.85)	114.25 (5.27)	120.31 (0.17)	113.21 (-)	115.55 (0.08)	0.1084
Length of the 5^th^ muscle attached to belly					42.21 (-)	43.41 (1.76)				0.8942
Height of the 5^th^ muscle belly					23.36 (3.82)	15.76 (7.40)	19.11 (0.01)	18.87 (-)	14.44 (0.18)	0.0605
Height of the 5^th^ muscle belly at 1/2 length					26.24 (5.68)	14.29 (6.26)	12.83 (0.08)	12.21 (-)	9.90 (0.12)	0.0275
Length of the 6^th^ muscle belly						119.03 (0.25)	121.88 (0.18)	119.23 (-)	123.00 (0.04)	0.1283
Height of the 6^th^ muscle belly						23.54 (0.15)	11.91 (0.05)	11.22 (-)	12.13 (0.02)	0.1283
Length of the 7^th^ muscle belly						20.42 (0.30)	8.88 0.01 (-)	87.21 (-)	10.57 (0.04)	0.1283
Height of the 7^th^ muscle belly							118.05 (0.11)	17.87 (-)	114.35 (0.52)	0.1653
Height of the 7^th^ muscle belly at 1/2 length							10.32 (0.01)	14.92 (-)	15.33 (0.01)	0.1653
Length of the 8^th^ muscle belly							6.16 (0.02)	98.87 (-)	6.68 (0.35)	0.1653
Height of the 8^th^ muscle belly								20.66 (-)	86.60 (0.53)	0.0010
Height of the 8^th^ muscle belly at 1/2 length								14.33 (-)	17.25 (0.08)	0.6254
Length of the 9^th^ muscle belly									15.46 (0.35)	—
Mean cross-sectional area	8758.66 (-)	6181.43 (1.52)	7621.12 (1190.27)	9307.43 (5682.75)	8326.01 (1854.78)	9686.83 (818.91)	8898.31 (19.36)	9300.11 (-)	9463.04 (3.79)	0.0223

## Data Availability

Please contact authors for data requests (Łukasz Olewnik PhD—email address: lukasz.olewnik@umed.lodz.pl).
